# Recalibration and validation of the Charlson Comorbidity Index in an Asian population: the National Health Insurance Service-National Sample Cohort study

**DOI:** 10.1038/s41598-020-70624-8

**Published:** 2020-08-13

**Authors:** Jae Shin Choi, Myoung-Hee Kim, Yong Chul Kim, Youn-Hee Lim, Hyun Joo Bae, Dong Ki Kim, Jae Yoon Park, Junhyug Noh, Jung Pyo Lee

**Affiliations:** 1 Department of Internal Medicine, Pyeongtaek St. Mary’s Hospital, Pyeongtaek-si, Gyeonggi-do Republic of Korea; 2 Department of Dental Hygiene, College of Health Science, Eulji University, Seongnam-si, Gyeonggi-do Republic of Korea; 3 Department of Internal Medicine, Seoul National University Hospital, Seoul, Republic of Korea; 4 Institute of Environmental Medicine, Seoul National University Medical Research Center, Seoul, Republic of Korea; 5 Future Environmental Strategy Research Group, Korea Environment Institute, Sejong-si, Republic of Korea; 6 Department of Internal Medicine, Dongguk University Ilsan Hospital, Goyang-si, Gyeonggi-do Republic of Korea; 7 Computer Science and Engineering, College of Engineering, Seoul National University, Seoul, Republic of Korea; 8 Department of Internal Medicine, Seoul National University Boramae Medical Center, Seoul, Republic of Korea; 9 Department of Internal Medicine, Seoul National University College of Medicine, Seoul, Republic of Korea

**Keywords:** Diseases, Health care

## Abstract

Weights assigned to comorbidities in predicting mortality may vary based on the type of index disease and advances in the management of comorbidities. We aimed to develop a modified version of the Charlson Comorbidity Index (CCI) using an Asian nationwide database (mCCI-A), enabling the precise prediction of mortality rates in this population. The main data source used in this study was the National Health Insurance Service-National Sample Cohort (NHIS-NSC) obtained from the National Health Insurance database, which includes health insurance claims filed between January 1, 2002, and December 31, 2013, in Korea. Of the 1,025,340 individuals included in the NHIS-NSC, 570,716 patients who were hospitalized at least once were analyzed in this study. In total, 399,502 patients, accounting for 70% of the cohort, were assigned to the development cohort, and the remaining patients (n = 171,214) were assigned to the validation cohort. The mCCI-A scores were calculated by summing the weights assigned to individual comorbidities according to their relative prognostic significance determined by a multivariate Cox proportional hazard model. The modified index was validated in the same cohort. The Cox proportional hazard model provided reassigned severity weights for 17 comorbidities that significantly predicted mortality. Both the CCI and mCCI-A were correlated with mortality. However, compared with the CCI, the mCCI-A showed modest but significant increases in the ***c*** statistics. According to the analyses using continuous net reclassification improvement, the mCCI-A improved the net mortality risk reclassification by 44.0% (95% confidence intervals (CI), 41.6–46.5; *p* < 0.001). The mCCI-A facilitates better risk stratification of mortality rates in Korean inpatients than the CCI, suggesting that the mCCI-A may be a preferable index for use in clinical practice and statistical analyses in epidemiological studies.

## Introduction

Comorbidity is among the most important predictors of inpatient outcomes^1^. Among the various methods used to predict mortality by weighting comorbidities, the Charlson Comorbidity Index (CCI) has been widely utilized by health researchers to measure the burden of diseases. In 1984, Charlson et al. identified the clinical conditions to be included in the index by reviewing 559 hospital charts of patients admitted to medical services at a single hospital and then assessed the associations of these comorbidities with the 1-year all-cause mortality^2^.

The ability of this index to predict mortality has been validated in various disease subgroups, including cancer^3^, renal disease^4^, stroke^5^ and intensive care^6^. Nevertheless, there are several reasons to recalibrate and subsequently validate this index with inpatients to obtain better predictions of their mortality rates. First, because the management of inpatients and their comorbidities has advanced significantly over the past 30 years, the contribution of comorbidities to the mortality rate has likely also changed. Second, this index is not suitable for predicting long-term outcomes in general hospitalized patients because in the development of the CCI, only a small sample and the one-year mortality rate were considered. Therefore, this index should be recalibrated and revalidated using more recent data.

The recalibration and validation of comorbidities indices have been performed in various disease group such as the index of coexistent disease^7^, Davies index^8^, Khan index^9^, a modified version of the CCI in incident hemodialysis patients (mCCI-IHD)^10^ and a modified version of the CCI in incident peritoneal dialysis patients (mCCI-PD)^11^. However, indices created from these specific disease groups may not be optimal to use as a tool to predict the outcomes of generalized patients.

Therefore, the present study aimed to develop a modified version of the Charlson Comorbidity Index (mCCI-A) using an Asian nationwide database that reflected the recent changes of mortality due to the development of medical technology and the difference in prevalence of diseases from racial differences and to compare its performance with the original CCI.

## Results

### Baseline characteristics of the entire cohort

The baseline characteristics of the entire cohort, development cohort and validation cohort are listed in Table 1 of the 570,716 participants, 46.3% of the patients were men. The overall mortality rate was 3.83%.

**Table 1:** Baseline characteristics of the development and validation cohort.

Variables	Entire cohort (N = 570,716)	Development cohort (N = 399,502)	Validation cohort (N = 171,214)
**Age (N (%))**			
< 60 years	421,229 (73.81)	294,740 (73.78)	126,489 (73.88)
60–79 years	119,149 (20.88)	83,505 (20.9)	35,644 (20.82)
≥ 80 years	30,338 (5.32)	21,257 (5.32)	9,081 (5.3)
Male (N (%))	264,092 (46.27)	184,891 (46.28)	79,201 (46.26)
No. of deaths (N (%))	21,868 (3.83)	15,308 (3.83)	6,560 (3.83)
**Regional size by population**			
Metropolitan areas	250,785 (43.94)	175,852 (44.02)	74,933 (43.77)
City or rural areas	319,931 (56.06)	223,650 (55.98)	96,281 (56.23)
**Family income ratio**^a^			
0 (Medical aid)	19,778 (3.47)	13,854 (3.47)	5,924 (3.46)
1	40,195 (7.04)	28,283 (7.08)	11,912 (6.96)
2	38,107 (6.68)	26,850 (6.72)	11,257 (6.57)
3	37,959 (6.65)	26,493 (6.63)	11,466 (6.70)
4	41,679 (7.30)	29,233 (7.32)	12,446 (7.27)
5	46,622 (8.17)	32,499 (8.13)	14,123 (8.25)
6	53,477 (9.37)	37,500 (9.39)	15,977 (9.33)
7	60,295 (10.56)	42,032 (10.52)	18,263 (10.67)
8	70,504 (12.35)	49,440 (12.38)	21,064 (12.30)
9	79,311 (13.90)	55,522 (13.90)	23,789 (13.89)
10	82,789 (14.51)	57,796 (14.47)	24,993 (14.60)

^a^Family income ratio was divided into the following 11 groups: medical aid with group 0 and the income decile with 10 equal -sized groups according to the rank of the gross household income and registered National Health Insurance (based on 2010).

In total, 74.43% of the subjects had one or more comorbidities. Among the 17 comorbidities, the most prevalent comorbidity was chronic pulmonary disease (47.56%), followed by ulcer disease (37.1%) and mild liver disease (24.05%) (Table 2).

**Table 2:** Prevalence of 17 comorbidities in the development and validation cohorts.

Comorbidity	Entire cohort (N = 570,716)	Development cohort (70%) (N = 399,502)	Validation cohort (30%) (N = 171,214)
No comorbidity	145,929 (25.57)	102,046 (25.54)	43,883 (25.63)
Ulcer disease	211,737 (37.1)	148,101 (37.07)	63,636 (37.17)
Peripheral vascular disease	50,688 (8.88)	35,642 (8.92)	15,046 (8.79)
Mild liver disease	137,253 (24.05)	95,987 (24.03)	41,266 (24.1)
Myocardial infarct	10,170 (1.78)	7,129 (1.78)	3,041 (1.78)
Connective tissue disease	41,225 (7.22)	28,817 (7.21)	12,408 (7.25)
Congestive heart failure	27,970 (4.9)	19,582 (4.9)	8,388 (4.9)
Chronic pulmonary disease	271,438 (47.56)	190,206 (47.61)	81,232 (47.44)
Diabetes mellitus	69,665 (12.21)	48,668 (12.18)	20,997 (12.26)
Diabetes mellitus with end organ damage	35,200 (6.17)	24,644 (6.17)	10,556 (6.17)
Hemiplegia	10,154 (1.78)	7,132 (1.79)	3,022 (1.77)
Cerebrovascular disease	63,988 (11.21)	44,836 (11.22)	19,152 (11.19)
Dementia	16,524 (2.9)	11,580 (2.9)	4,944 (2.89)
Moderate or severe renal disease	7,494 (1.31)	5,274 (1.32)	2,220 (1.3)
Any tumor, leukemia, lymphoma	39,575 (6.93)	27,730 (6.94)	11,845 (6.92)
Moderate or severe liver disease	5,334 (0.93)	3,697 (0.93)	1,637 (0.96)
Metastatic solid tumor	12,410 (2.17)	8,664 (2.17)	3,746 (2.19)
AIDS	162 (0.03)	104 (0.03)	58 (0.03)
One or more comorbidity	424,787 (74.43)	297,456 (74.46)	127,331 (74.37)

### Development of the New Comorbidity Index (mCCI-A) for the prediction of mortality

**Figure 1:**
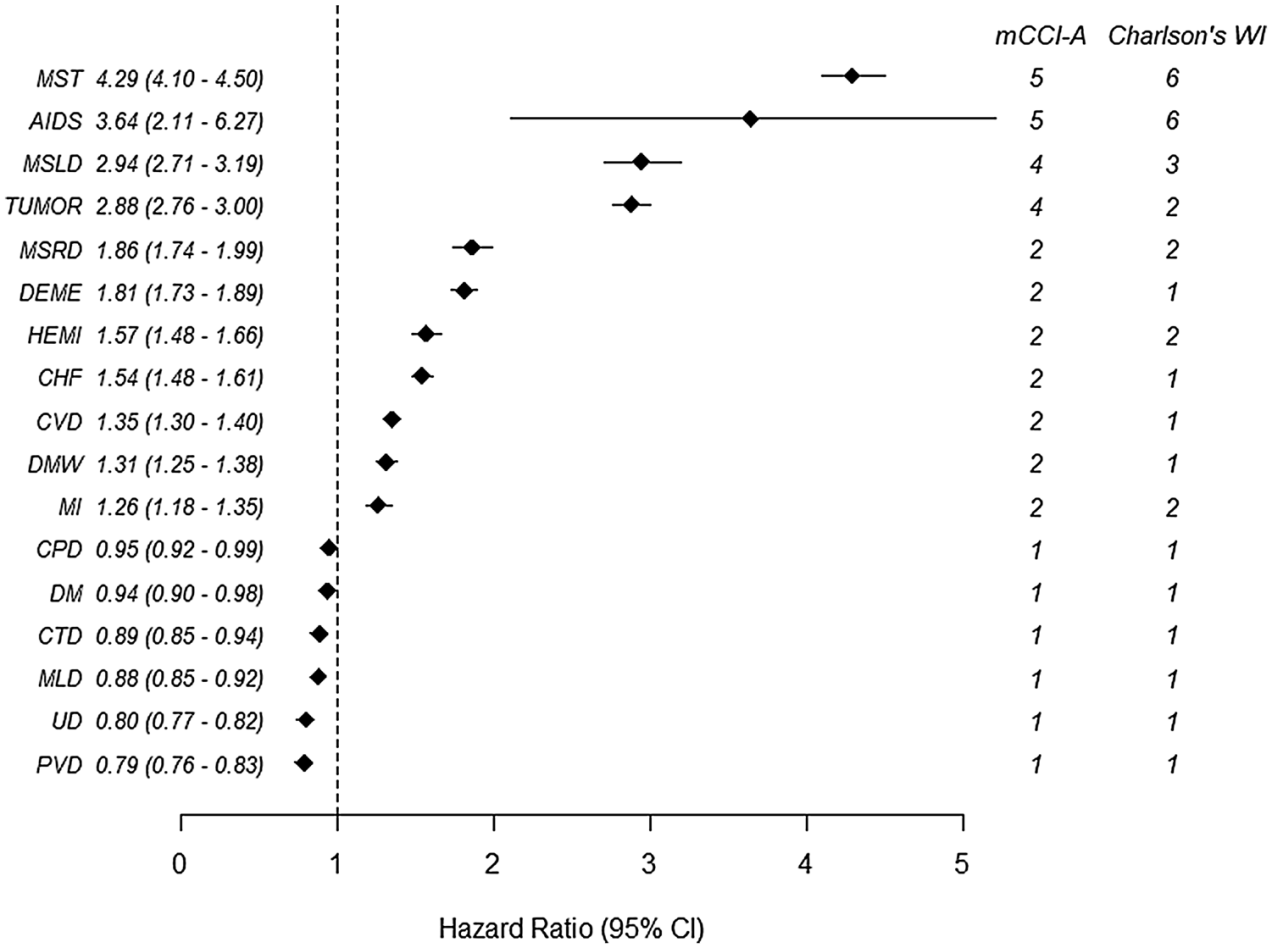
Adjusted hazard ratio and weights of 17 comorbidities in the development cohort. MST, Metastatic solid tumor; AIDS, Acquired immunodeficiency syndrome; MSLD, Moderate or severe liver disease; MSRD, Moderate or severe renal disease; DEME, Dementia; HEMI, Hemiplegia, CHF, Congestive heart failure, MI, Myocardial infarction; CVD, Cerebrovascular disease; DMW, Diabetes mellitus with end organ damage; DM, Diabetes mellitus; CPD, Chronic pulmonary disease; MLD, Mild liver disease; CTD, Connective tissue disease; UD, Ulcer disease; PVD, Peripheral vascular disease. Adjusted for age (quartile), sex, region, family income ratio (11 groups), and all comorbidities.

Figure 1 shows the adjusted hazard ratios (HRs) and weights of each comorbidity. All comorbidities were significantly associated with mortality. Metastatic solid tumors were assigned the highest weight, followed by AIDS, moderate or severe liver disease, and any tumor, consecutively (Table 3).

**Table 3:** Weights for comorbidities in the development cohort.

	Development cohort (N = 399,502)			
	HR (95% CI)^a^	*P* value	Relative weight	Weight
**Comorbidity**				
Peripheral vascular disease	0.79 (0.757–0.826)	< 0.001	1	1
Ulcer disease	0.80 (0.769–0.823)	< 0.001	1.013	1
Mild liver disease	0.88 (0.852–0.917)	< 0.001	1.114	1
Connective tissue disease	0.89 (0.850–0.941)	< 0.001	1.127	1
Diabetes	0.94 (0.896–0.976)	0.002	1.19	1
Chronic pulmonary disease	0.95 (0.921–0.985)	0.002	1.203	1
Myocardial infarct	1.26 (1.183–1.346)	< 0.001	1.595	2
Diabetes with end organ damage	1.31 (1.247–1.376)	< 0.001	1.658	2
Cerebrovascular disease	1.35 (1.297–1.399)	< 0.001	1.709	2
Congestive heart failure	1.54 (1.477–1.606)	< 0.001	1.949	2
Hemiplegia	1.57 (1.479–1.664)	< 0.001	1.987	2
Dementia	1.81 (1.727–1.887)	< 0.001	2.291	2
Moderate or severe renal disease	1.86 (1.740–1.986)	< 0.001	2.354	2
Any tumor, leukemia, lymphoma	2.88 (2.762–2.999)	< 0.001	3.646	4
Moderate or severe liver disease	2.94 (2.705–3.186)	< 0.001	3.722	4
AIDS	3.64 (2.110–6.269)	< 0.001	4.608	5
Metastatic solid tumor	4.29 (4.095–4.502)	< 0.001	5.43	5

^a^Adjusted for age (10 groups), sex, region, family income ratio, and all comorbidities.

Compared with the weights in the CCI, in the mCCI-A, the updated weights for cerebrovascular disease, myocardial infarction (MI), congestive heart failure (CHF), dementia, any tumor and moderate or severe liver disease increased; the updated weights for acquired immunodeficiency syndrome (AIDS) and metastatic solid tumors decreased; and the updated weights for diabetes without complication, diabetes with end organ damage, hemiplegia, and moderate or severe renal disease did not change (Fig. 1).

**Figure 2:**
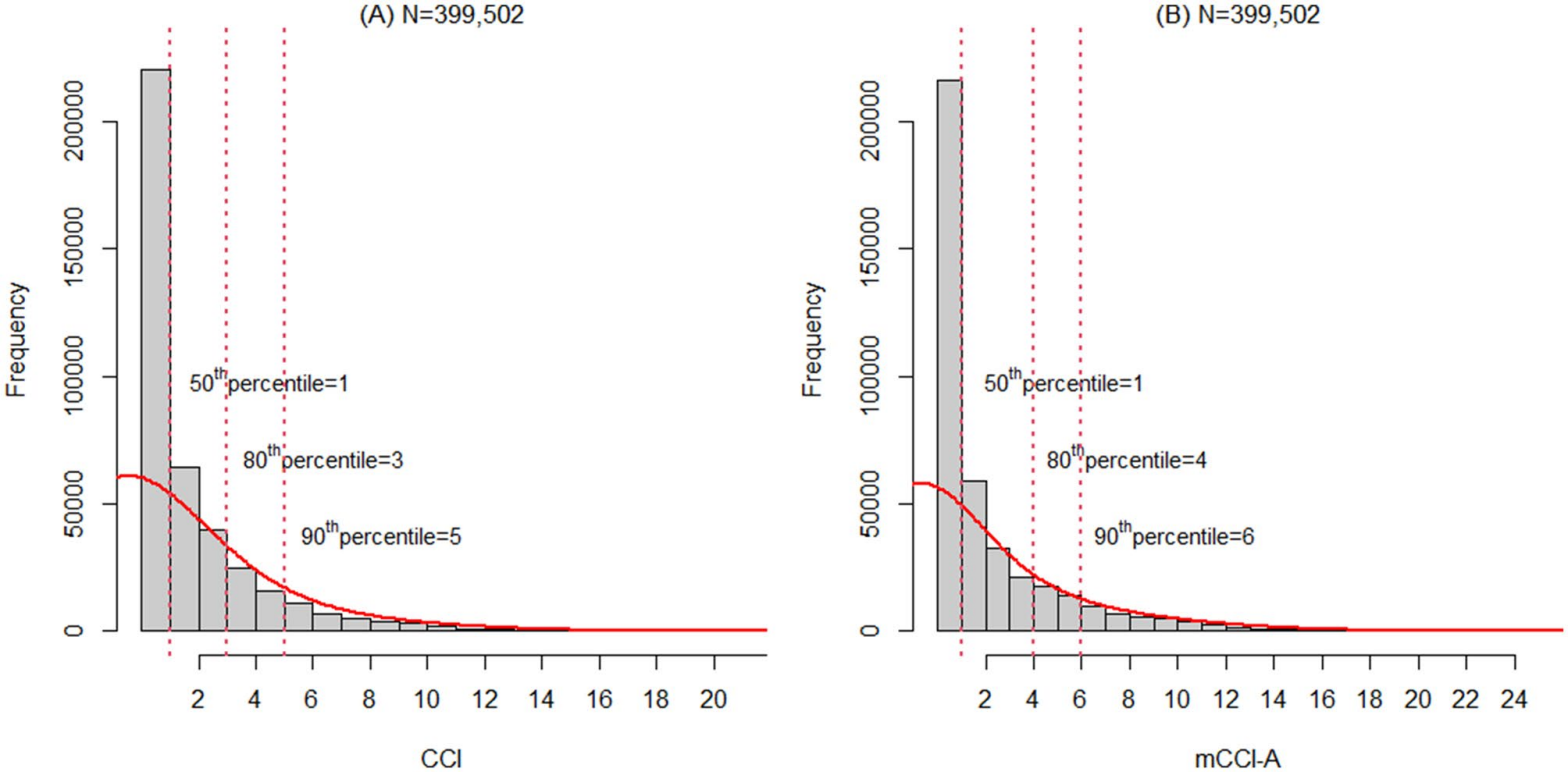
Distribution of the CCI and mCCI-A scores in the development cohort. (**A**) Distribution of the CCI scores (n = 399,502); (**B**) Distribution of the mCCI-A scores (n = 399,502). The y-axis shows the number of subjects. The solid line represents a density curve calculated by approximation to identify the overall pattern and deviation. The vertical dotted lines (red) represent the 50th, 80th, and 90th percentile values. CCI, Charlson Comorbidity Index; mCCI-A, modified version of the Charlson Comorbidity index for Asian populations.

The mCCI-A scores were calculated by summing the updated weights. The scores were applied to each patient in the development cohort. Based on the CCI and mCCI-A scores, we categorized both scores into the following 4 risk groups: ≤ 50th percentile, 50th–80th percentile, 80th–90th percentile and > 90th percentile. To determine the cut-off values of the comorbidity scores in each risk group, we generated a histogram to represent the distributions of the scores (Fig. 2). The cut-off values of CCI scores corresponding to the 50th, 80th and 90th percentiles were 1, 3 and 5, respectively, whereas those of the mCCI-A scores were 1, 4 and 6, respectively.

**Figure 3:**
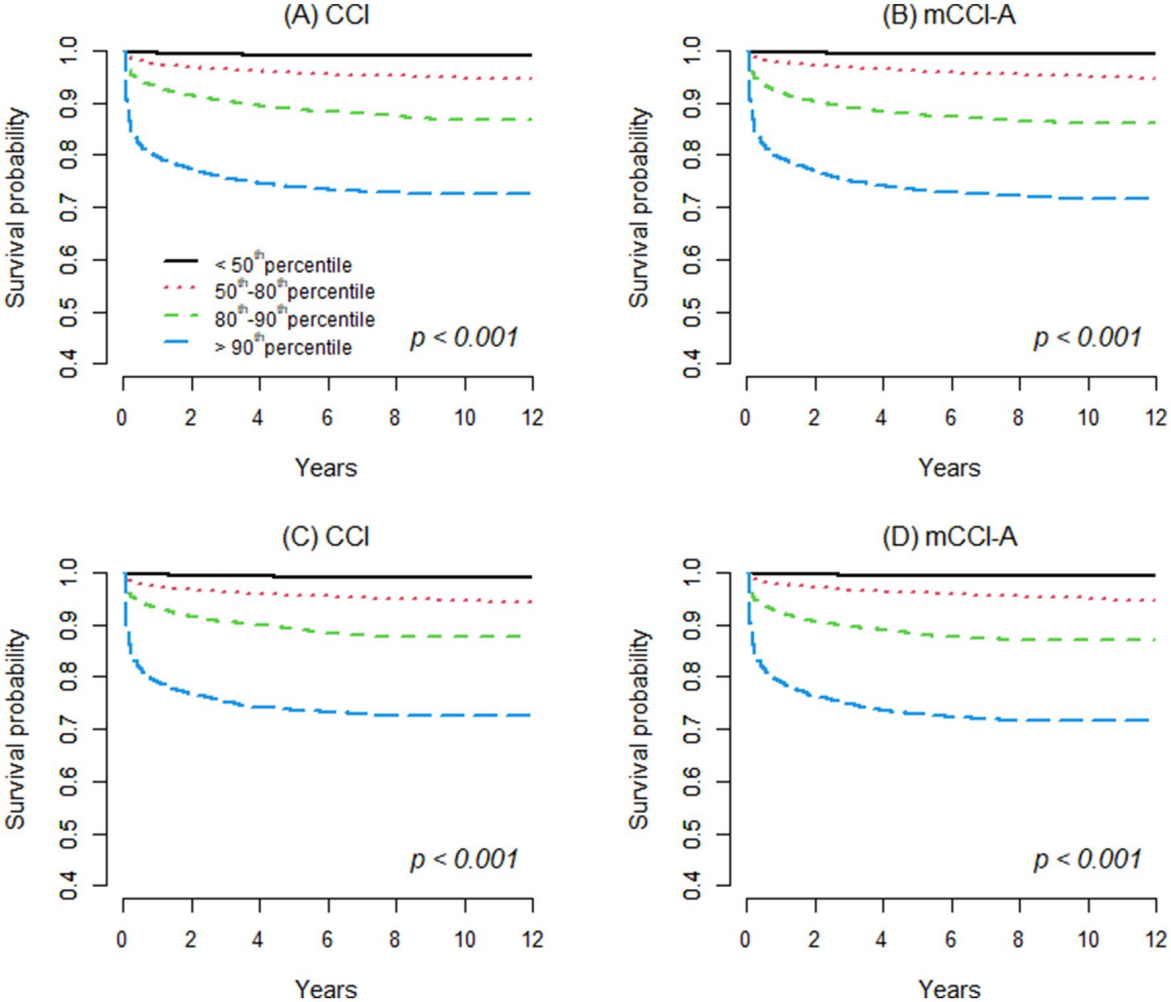
Survival curves obtained using the Kaplan–Meier method in the development and validation cohorts differentiated by 4 risk groups for CCI and mCCI-A. (**A**) Survival curves in the development cohort for CCI, (**B**) Survival curves in the development cohort for mCCI-A, (**C**) Survival curves in the validation cohort for CCI, (**D**) Survival curves in the validation cohort for mCCI-A. CCI in development cohort, < 50th percentile (n = 220,258, scores 0–1); 50th–80th percentile (n = 103,849, scores 2–3); 80th–90th percentile (n = 40,558, scores 4–5) and > 90th percentile (n = 34,837, score ≥ 6). mCCI-A in the development cohort, < 50th percentile (n = 215,768, scores 0–1); 50th–80th percentile (n = 113,413, scores 2–4); 80th–90th percentile (n = 31,535, scores 5–6) and > 90th percentile (n = 38,786, score ≥ 7). CCI in the validation cohort, < 50th percentile (n = 94,378, scores 0–1); 50th–80th percentile (n = 44,517, scores 2–3); 80th–90th percentile (n = 17,467, scores 4–5) and > 90th percentile (n = 14,852, score ≥ 6). mCCI-A in the validation cohort, < 50th percentile (n = 92,507, scores 0–1); 50th–80th percentile (n = 48,490, scores 2–4); 80th–90th percentile (n = 13,626, scores 5–6) and > 90th percentile (n = 16,591, score ≥ 7). CCI, Charlson Comorbidity Index; mCCI-A, modified version of the Charlson Comorbidity Index for Asian populations.

Figure 3 shows the survival curves obtained using the Kaplan–Meier method in the development cohort, which were differentiated by the 4 risk groups of the CCI and mCCI-A. The risk groups in each index could discriminate the survival rates, indicating that increasing comorbidity index scores were associated with lower cumulative survival.

### Application and validation of the mCCI-A

The baseline characteristics of the validation cohort (n = 171,214) are listed in Table 1. Males represented 46.26% of the subjects. The overall mortality was 3.83%. In total, 74.37% of the subjects had one or more comorbidities. Among the 17 comorbidities, the most prevalent comorbidity was chronic pulmonary disease (47.44%), followed by ulcer disease (37.17%) and mild liver disease (24.1%) (Table 2).

To assess the discrimination ability of each index, the *c* statistic and cNRI were calculated after adjusting for confounders in the following two analyses: univariate and multivariate. Significant differences were observed in the *c* statistics between the CCI and the mCCI-A in the univariate and multivariate analyses (Table 4). Additionally, a significant risk reclassification improvement was observed in the mCCI-A in the univariate and multivariate analyses using cNRI. In the multivariate analysis, compared with the CCI, the mCCI-A significantly improved the net mortality risk reclassification by 44.0% (95% CI 41.6–46.5; *p* < 0.001), indicating that the mCCI-A facilitates a better risk stratification of mortality in Korean inpatients than the CCI. Figure 3 shows the survival curves obtained using the Kaplan–Meier method in the validation cohort, which were differentiated by the 4 risk groups of the CCI and mCCI-A.

**Table 4:** Model performance of the mCCI-A in the validation and development cohorts.

	*c* statistic (95% CI)	*p*-value^a^	cNRI_Event_ (%, 95% CI)	*p-* value	cNRI_Non-event_ (%, 95% CI)	*p*-value	cNRI_Total_ (%, 95% CI)	*p*-value
**Validation cohort (n = 171,214)**								
Model 1								
CCI	0.831 (0.826–0.836)							
mCCI-A	0.855 (0.850–0.860)	< 0.001	16.6 (14.2–18.9)	< 0.001	71.1 (70.8–71.5)	< 0.001	87.7 (85.3–90.1)	< 0.001
Model 2								
CCI	0.890 (0.887–0.893)							
mCCI-A	0.898 (0.895–0.901)	< 0.001	9.9 (7.4–12.3)	< 0.001	34.2 (33.7–34.6)	< 0.001	44.0 (41.6–46.5)	< 0.001
**Development cohort (n = 399,502)**								
Model 1								
CCI	0.827 (0.824–0.830)							
mCCI-A	0.851 (0.848–0.854)	< 0.001	15.0 (13.5–16.6)	< 0.001	70.9 (70.7–71.1)	< 0.001	85.9 (84.3–87.5)	< 0.001
Model 2								
CCI	0.888 (0.886–0.890)							
mCCI-A	0.895 (0.894–0.897)	< 0.001	8.7 (7.1–10.3)	< 0.001	31.2 (30.9–31.5)	< 0.001	39.9 (38.3–41.5)	< 0.001

cNRI, continuous net reclassification improvement; CCI, Charlson Comorbidity Index; mCCI-A, modified version of the Charlson Comorbidity Index for Asian populations; cNRI_Total_ = cNRI_Event_ + cNRI_Non-event_.

Model 1; univariate. Model 2; adjusted for age (quartile), sex, region and family income ratio (11 groups).

^a^*p*-value for the *c* statistic was taken and computed for a contrast test.

### Application and validation of the mCCI-A to specific diseases

To assess the discrimination ability of each index, the *c* statistic and cNRI were calculated after adjusting for confounders in multivariate analysis in subgroups such as mild liver disease, chronic pulmonary disease, diabetes mellitus and moderate/severe renal disease patients composed of the total cohort. Significant differences were observed in the *c* statistics and cNRI between the CCI and the mCCI-A in multivariate analyses (Table 5).

**Table 5:** Model performance of the mCCI-A in the subgroup analysis with specific disease.

	*c* statistic (95% CI)	*p*-value^a^	cNRI_Event_ (%, 95% CI)	*p-* value	cNRI_Non-event_ (%, 95% CI)	*p*-value	cNRI_Total_ (%, 95% CI)	*p*-value
**Mild liver disease (n = 137,253)**								
CCI	0.851 (0.846–0.855)							
mCCI-A	0.862 (0.853–0.866)	< 0.001	4.8 (2.3–7.2)	< 0.001	43.8 (43.3–44.3)	< 0.001	48.5 (46.1–51.0)	< 0.001
**Chronic pulmonary disease (n = 271,438)**								
CCI	0.878 (0.876–0.880)							
mCCI-A	0.887 (0.885–0.889)	< 0.001	9.3 (7.5–11.1)	< 0.001	35.3 (34.9–35.6)	< 0.001	44.6 (42.8–46.4)	< 0.001
**Diabetes mellitus (n = 69,665)**								
CCI	0.753 (0.747–0.759)							
mCCI-A	0.768 (0.763–0.774)	< 0.001	6.6 (4.2–9.1)	< 0.001	32.6 (31.8–33.3)	< 0.001	39.2 (36.6–41.8)	< 0.001
**Moderate or severe renal disease (n = 7,494)**								
CCI	0.665 (0.651–0.679)							
mCCI-A	0.676 (0.662–0.691)	< 0.001	14.8 (9.7–19.8)	< 0.001	20.7 (18.3–23.2)	< 0.001	35.5 (29.8–41.1)	< 0.001

cNRI, continuous net reclassification improvement; CCI, Charlson Comorbidity Index; mCCI-A, modified version of the Charlson Comorbidity Index for Asian populations; cNRI_Total_ = cNRI_Event_ + cNRI_Non-event_.

All models were adjusted for age (quartile), sex, region and family income ratio (11 groups).

^a^*p*-value for the *c* statistic was taken and computed for a contrast test.

## Discussion

In this study, we modified the CCI using a nationwide population-based database and to developed a comorbidity index that provides better risk predictions in the general inpatient population. To the best of our knowledge, this study is the largest study to modify the CCI using a nationwide population-based database and develop a comorbidity index that provides better risk prediction in a general inpatient Asian population.

Since 1987, the CCI has been used as a comorbidity index throughout the medical community, and there have been efforts to predict the outcome of the general inpatient population using the CCI since the 1980s to 2000s. In 1996, the CCI was applied to 33,940 inpatients with ischemic heart disease^12^. In 1992, another study involving 27,111 patients who underwent lumbar spine surgery was conducted^13^. In both of the above mentioned studies, the diagnostic information used was based on International Classification of Disease, 9th revision codes (ICD-9). Using ICD codes may be useful in exploratory data analyses^14^. In the 2000s, a retrospective cohort study was conducted using National Health Insurance claims data (2001–2002) to compare the performance of three comorbidity measurements (Elixhauser, Charlson/Deyo, and Charlson/Romano method) among inpatients hospitalized for Chronic Obstructive Pulmonary Disease and Acute Myocardial Infarction in Taiwan^15^. However, these studies had two limitations. First, these studies investigated specific disease groups rather than general patients. Second, the purpose of these studies was to demonstrate superiority among the existing methods or to apply the methods to the general population.

Previous studies have directly applied the CCI to patients using a weight equal to that in the original index for each CCI index. However, the recalibration and validation of the weights of the CCI index diseases are needed for several reasons. First, the original CCI does not reflect the significant progress achieved in the treatment of each comorbidity and medical advancements over the previous 30 years. Second, the extent to which the original CCI reflects long-term outcomes considered important is unclear because the “training” population used to develop the original CCI was created based on the one-year mortality rate in general inpatients. Third, the original CCI was developed based on a relatively small number of patients.

In 1996, a study was conducted to update the CCI and scores using USA administrative databases of 6,326 patients who underwent bypass surgery, and the new index exhibited superior performance over the original CCI (c = 0.74 vs. 0.70)^16^. A new comorbidity index was developed by assigning specific weights to the original CCI in this study. However, AIDS was excluded because no patients with AIDS were included in the sample. In 2011, a study updated the CCI and scores using Canadian administrative databases of 55,929 patients admitted to a medical facility in the Calgary region (Alberta, Canada) (population 1.3 million)^17^. The authors excluded the 5 comorbidities found to have no statistical correlation with the mortality rates among the 17 comorbidities. The new index exhibited superior performance over the original CCI (c = 0.825 vs. 0.808). Although these two studies reflect the recent advances in medical management, there are limitations in that study. The evaluation of several comorbidities was limited (particularly AIDS), and there is still a need for further evidence regarding whether the index can be directly applied to Asians.

In Asia, the CCI has also been applied to various disease groups to evaluate the various outcomes of patients. However, the usefulness of the original CCI remains controversial. A Japanese study revealed that the CCI had been used to predict the overall survival of patients with solid tumors; however, the CCI is not considered a significant predictor^18^. The authors emphasized the necessity for developing scales that can more accurately predict patient outcomes.

Previously, we updated the CCI and scores in hemodialysis and peritoneal dialysis patients using the National Health Insurance dataset in Korea^10,11^. The first study involved 24,738 people who first started hemodialysis between 2005 and 2008^10^. We developed the mCCI-IHD, which included 14 comorbidities with reassigned severity weights. In the validation cohort, compared with the CCI, the mCCI-IHD showed modest but significant increases in the *c* statistics at 6 months and 1 year. Compared with the CCI, the analyses using cNRI revealed that the mCCI-IHD improved the net mortality risk reclassification by 24.6%, 26.2% and 42.8% at 6 months and 1 and 2 years, respectively. The second study involved 7,606 people who first started peritoneal dialysis between 2005 and 2008^11^. We developed the mCCI-IPD, which included 11 comorbidities with reassigned severity weights. In the validation cohort, compared with the CCI, although the mCCI-IHD showed no differences in the *c* statistics, the analyses using cNRI revealed that the mCCI-IHD provided a 38.2% improvement in mortality risk assessment.

Based on the results of the previous two studies, we extended this study to the entire inpatient population. In this study, we modified the CCI using administrative data that included nearly all Korean inpatients who were admitted between 2002 and 2013 and developed a comorbidity index that provides better risk stratification. In addition, we performed a cross validation of the new index. Additionally, the superior performance of the new index over the original CCI was shown. Notably, this report has two major implications. First, in contrast to the previous CCI, the mCCI-A score reflects the current medical environment. Comparing the weights in the CCI with the weights in the mCCI-A, the increased weights of cerebrovascular disease, circulatory systems (MI and CHF) and nonmetastatic tumors are likely associated with the increased prevalence of these diseases. This tendency is consistent with recent studies investigating populations with specific diseases in Korea^10,11^. Additionally, recent advances in the effectiveness of the treatment of metastatic cancer has led to a decreased mortality rate. This change is likely due to the decreased weights of metastatic cancer in this study. The second implication is that this study, based on the overall cohort of inpatients, overcame the limitations associated with the use of small sample sizes in previous studies. For example, AIDS was not considered in a previous study modifying the CCI due to the small sample size. However, in this study, obtaining the weights of AIDS was possible because of the large sample size.

This study has several limitations. First, in contrast to collecting data through chart reviews, the determination of the prevalence of diseases is generally problematic using administrative data^19^. Second, although the use of administrative data has advantages, such as the conservation of time and resources and consistency in diagnosis, the diagnoses may be inappropriate due to physician preferences regarding diagnostic codes. Third, administrative data do not include biochemical parameters such albumin and hemoglobin, which could affect the survival rate. Fourth, new values for the weight of each comorbidity were derived in this study, but no expert agreement on them could be derived and will need to be considered later. Fifth, because this study was conducted for patients from 2002 to 2013, there is a limitation to the application of hospitalized patients after 2013. Last, although there were interactions between variables and comorbidities that we considered in our analysis, the interaction test was not conducted in our study.

## Methods

### Data source and study samples

The main data source used in this study was the National Health Insurance Service-National Sample Cohort (NHIS-NSC) obtained from the National Health Insurance Service (NHIS) in South Korea. The NHIS-NSC database has been publicly available since July 2014 and contains a substantial amount of information regarding health care utilization, health screening, sociodemographic variables and mortality in Korean citizens. Detailed information obtained from the NHIS-NSC has been previously published^20,21^. Briefly, The NHIS-NSC covers health insurance claims filed between January 1, 2002 and December 31, 2013. Of the eligible population in 2002 (46,605,433 target individuals of 47,851,928 individuals constituting the entire Korean population), 1,025,340 participants (2.2% of the target population) were randomly selected until 2013 and were followed until 2013 (for 12 years). All traceable identifiers were removed before publishing to protect patient confidentiality.In this study, 578,547 patients (56.4% of the NHIS-NSC) who were hospitalized at least once were analyzed longitudinally. Except for 7,831 patients who died immediately or same month as hospitalization after admission, the total number of patients in our cohort was 570,716.

### Study variables

Using the International Classification of Diseases, 10th revision (ICD-10), we identified the Charlson comorbidities among the secondary diagnoses based on all diseases that were diagnosed from both inpatient and outpatient services before discharge^22^. To develop the modified index, we used the same comorbid conditions covered by the CCI, including MI, CHF, peripheral vascular disease, cerebrovascular disease, dementia, chronic pulmonary disease, connective tissue disease, ulcer disease, mild liver disease, diabetes, hemiplegia, diabetes with end-organ damage, any tumor (including leukemia and lymphoma), moderate to severe liver disease, metastatic solid tumors and AIDS. We retrieved all records of each patient from the National Health Insurance database prior to the date of the discharge to identify the comorbidities. A patient was considered to have a comorbid condition if the condition was present in the index admission records. The outcomes included all-cause long-term mortality within the follow-up period after admission. Additionally, the National Health Insurance claims databases were used to identify mortality. Death occurring between January 1, 2002 and December 31, 2013, was considered in the development and validation cohorts. However, patients with admission and death occurring in the same month were excluded. The demographic information of both cohorts included age, sex, region, and family income.

### Statistical analysis

First, to analyze the baseline characteristics of the study population, we merged the demographic and medical utilization data. A Cox regression analysis adjusted for age (quartile), sex, region and family income ratio was performed to develop new weights for the comorbidities. We obtained the adjusted HRs and 95% confidence intervals (CIs) after adjusting for age, sex, region, family income ratio, and all 17 comorbidities. The prognostic weights of the mCCI-A were computed by dividing the HRs associated with each comorbidity by the lowest HRs^23^. Then, the relative weights were truncated to integer values rounded to zero decimal places. The comorbidity score of each patient was calculated by summing the weights. Kaplan–Meier survival curves^24^ were generated to compare the performance of the CCI to the performance of the mCCI-A.

We performed internal-validation partitioning of the main data set into two sets comprising 70% of the sample for training and 30% of the sample for testing using a random sampling function with the variable of death as a reference parameter. In total, 70% (n = 399,502) of the sample was used for training to develop new comorbidity weights, and the remaining 30% (n = 171,214) of the sample was used as a validation cohort.

To assess the capacity of discriminating between the indices, a *c* statistic was calculated using the area under the receiver-operator curve^25^. To determine the statistical significance and 95% CIs of the *c* statistic, a Mann–Whitney test was performed with a contrast test. The continuous net reclassification improvement (cNRI) score obtained by performing logistic regression models was calculated to evaluate the reclassification^26^. For the binary response, i.e., death, the function *improveprob* in R was used to determine whether the predictions obtained from the model of mCCI-A significantly differed from those obtained from the model of the original CCI^27^. The cNRI_total_ is the sum of cNRI_Event_ and cNRI_Non-event_, indicating the sum of the net proportions of subjects who died (Event) and who did not die (Non-event) were correctly reassigned a predicted risk.

The data were analyzed by using SAS 9.4 for Windows software (SAS Institute, Cary, NC, USA) and R software version 4.0.0 (Comprehensive R Archive Network: https://cran.r-project.org). In all analyses, *p* < 0.05 was considered statistically significant.

### Ethical approval

This study was conducted in accordance with the principles of the Declaration of Helsinki. The study was approved by the Institutional Review Board (IRB) of Seoul National University Hospital (1610-038-797), and the study protocol was approved by the IRB. Under IRB approval, the informed consent waived.

## Data Availability

The datasets generated during and/or analyzed during the current study are available from the corresponding author on reasonable request.
